# Experimental Study on the Compressive Behavior of Fiber-Reinforced Ceramsite Concrete

**DOI:** 10.3390/ma18040862

**Published:** 2025-02-16

**Authors:** Fei Gu, Congqi Li, Xin Wang, Yang Yang, Hushan Liu

**Affiliations:** 1 College of Architectural Science and Engineering, Yangzhou University, Yangzhou 225127, China; mx120220605@stu.yzu.edu.cn (F.G.); mz120221076@stu.yzu.edu.cn (X.W.); 2 Jiangsu Hetianxia Energy Saving Technology Co., Ltd., Yangzhou 225012, China; jshtx9@163.com

**Keywords:** fiber-reinforced ceramsite concrete, preparation process, compression performance, stress–strain relationship model

## Abstract

Ceramsite concrete is a kind of green building material with advantages such as low weight, heat insulation, and fire resistance. However, it has low strength, high brittleness, and the problem of aggregate floating. In this study, by adding polypropylene fibers and optimizing the preparation process, the mechanical properties of ceramsite concrete have been significantly improved, which is of great significance for promoting the application of this material in the engineering field. Through uniaxial compressive tests on 54 specimens in six groups (divided into three strength grades), the failure characteristics and stress–strain relationships of each group of specimens were analyzed, and the effects of strength grades and fiber contents on parameters such as peak stress, peak strain, ultimate strain, and elastic modulus were studied. The results show that the addition of polypropylene fibers can improve the strength of ceramsite concrete, effectively improve the deformation performance and ductility of specimens before failure, and reduce brittleness. Specifically, as the fiber content increases, the peak stress first increases and then decreases, reaching its peak at a content of 0.05%, with an increase of 8.98%. At the same time, as the fiber content increases, the peak strain and ultimate strain increase significantly, reaching their peaks at a content of 0.075%, with increases of 21.3% and 25.2%, respectively. In addition, this paper proposes a piecewise correction model for the uniaxial compressive stress–strain curve of fiber-reinforced ceramsite concrete. This model has a good fit with the full experimental curve, providing an accurate theoretical reference for the application and development of this material in engineering.

## 1. Introduction

Ceramsite concrete is a kind of lightweight aggregate concrete (hereinafter referred to as LAC), which is prepared by replacing the coarse aggregate in traditional concrete with ceramsite. It has the following performance advantages: light weight and dry apparent density lower than 1950 kg/m^3^; the pore structure of ceramsite results in a “micro pump” effect within the concrete, and the dense interface between the aggregate and cementitious material effectively improves the frost resistance, impermeability, corrosion resistance, and durability of ceramsite concrete [1]. The internal structure of ceramsite concrete is rough and porous, leading to a low thermal conductivity. It has a good application prospect in prefabricated components such as external wall panels and roof panels of prefabricated buildings [2].

However, compared with traditional concrete, ceramsite concrete still has some drawbacks in engineering applications. This is mainly because the strength of ceramsite itself is low. When subjected to external forces, microcracks are likely to form inside the ceramsite, and these cracks can rapidly expand and connect, breaking through the interface between the ceramsite and the cement paste, leading to the failure of the overall structure [3]. This brittle behavior severely restricts the application of ceramsite concrete. In seismic design, it cannot effectively absorb and dissipate seismic energy like traditional concrete. Moreover, brittle failure occurs without warning, which greatly reduces the structural reliability and safety. China’s lightweight aggregate concrete structure design specification [4] and the American ACI 318 Code [5] have both specified the strength limit conditions for the use of ceramsite concrete in high-intensity seismic areas, that is, the strength grade should not exceed LC40. At the same time, due to the low density of ceramsite itself, the aggregates of ceramsite concrete tend to float during the mixing and vibrating processes, which complicates the control of the uniformity of its mixture.

In order to improve the homogeneity of a lightweight aggregate concrete mixture, domestic and foreign scholars have carried out corresponding research on its preparation process. Jianqing Gong et al. [6] added shrinkage reducer (SRA) to vitrified concrete and found that SRA can effectively reduce the drying self-shrinkage of concrete when the content of SRA is 3%; at the same time, the lower the water–cement ratio is, the better the drying shrinkage inhibition effect is. Yu. E. Pivinskii et al. [7] optimized the HCBS and vitrified concrete technology by using thinning, plasticizing, and reinforcing additives, and found that the highly effective modifiers achieved significant improvements in density and strength while increasing the density and strength of vitrified concrete at 7 d, 14 d, and 28 d. However, how to optimize the preparation process and improve the homogeneity of a LAC mixture based on the whole process of ceramsite concrete preparation remains to be further studied.

Because the fiber is distributed in three-dimensional chaos inside the cement-based mixture, it is well bonded to the matrix, which can effectively improve the deformation performance of the concrete. Domestic and foreign scholars have paid attention to the influence of different fiber types and fiber content on the basic mechanical properties and brittleness of LAC. Feng Shi et al. [8,9] significantly improved the mechanical properties of lightweight aggregate concrete by adding hybrid fibers containing basalt fibers (BF) and polypropylene fibers (PPF), which showed that BF and PPF were not only good for filling the internal pores of lightweight aggregate but also easily bond with cement mortar and improve the densification at the interface between the aggregate and cement transition zone. Lin H et al. [10] added 0%, 0.3%, 0.6%, and 0.9% high-tenacity polypropylene (HTPP) fibers into vitrified concrete. The test results showed that the HTPP fibers could significantly improve the axial compressive strength and toughness of ceramic concrete; the theoretical expressions for the stress–strain curves of the HTPP fiber-reinforced ceramic concrete were proposed after fitting the experimental curves. However, the advantages of steel fiber’s high strength and high elastic modulus cannot be fully utilized in lightweight aggregate concrete; its high cost affects its wide application in lightweight aggregate concrete [11]. Conversely, new synthetic low elastic modulus fibers like polypropylene fibers have certain advantages in the preparation of lightweight aggregate concrete prefabricated components due to their light weight, low cost, good ductility, good toughening and crack resistance, heat resistance, and acid and alkali corrosion resistance. At present, the influence of polypropylene fiber on the mechanical properties of LAC has attracted the attention of scholars at home and abroad. The stress–strain relationship and numerical model of polypropylene fiber lightweight aggregate concrete under uniaxial compression need to be further studied.

To clarify the influence of polypropylene fibers (PPF) on the basic mechanical properties of ceramsite concrete, improve the deformation performance of ceramsite concrete, and meet the requirements of engineering practice for the mechanical and workability properties of lightweight aggregate concrete (LAC), this paper proposes the mix proportions of fiber-reinforced ceramsite concrete with three strength grades based on comparative tests. The preparation process is optimized to suppress the floating of ceramsite, and fiber-ceramsite concrete with good uniformity is prepared. Axial uniaxial compressive tests are carried out on a total of 54 fiber-reinforced ceramsite concrete specimens in six groups to investigate the influence of PPF content on the compressive properties of ceramsite concrete with different strength grades. Special attention is paid to the changes in characteristic points on the stress–strain curve, including the initial elastic modulus, secant modulus, peak stress, peak strain, and ultimate strain. The influence of PPF content on its brittleness is analyzed, and a uniaxial compression constitutive model of PPF-ceramsite concrete is further proposed. This provides a reference for promoting the application of LAC in civil engineering and achieving the refined design of structures.

## 2. Research Preparation Process

In order to study the effect of PPF fiber admixture on the uniaxial compression stress–strain full curve and damage morphology, three kinds of fiber-reinforced vitrified concrete with strength grades LC20, LC30, and LC40 to meet the requirements were prepared, and the optimal mix design was carried out by referring to the ”Lightweight Aggregate Concrete Technical Specification” [12] with the total amount of cementitious materials, sand rate, water–cement ratio, mineral admixture, and fiber admixture as the influencing factors to design the optimum mix ratio.

### 2.1. Test Raw Materials

(1) Cement

The cement is P.O 42.5 Portland cement. The initial setting time and final setting time are 130 min and 220 min, respectively. The apparent density is 3100 kg/m^3^. The 80 μm sieve residue is 0.68%. The standard consistency of water is 26.3%. The 28d compressive strength and flexural strength are 54.8 MPa and 12.1 MPa, respectively.

(2) Coarse aggregate

Gravel aggregate, due to its substantial specific surface area and increased contact area with the slurry, generates greater viscous forces when moving within the slurry, thereby mitigating aggregate floating to a certain extent [13]. Therefore, gravel shale ceramsite is selected. LC20 and LC30 adopt 700 grade shale ceramsite, whose bulk density and dry apparent density are 497 kg/m^3^ and 820 kg/m^3^, respectively, and the cylinder compressive strength is 3.5 MPa. LC40 adopts 900 grade shale ceramsite, with the bulk density of 975 kg/m^3^, the dry apparent density of 1739 kg/m^3^, and the cylinder compressive strength of 6.5 MPa. The control of aggregate particle size is essential, as excessively large particles can result in serious mixture segregation, while overly small particles can increase the amount of cement slurry enveloping the aggregate, impacting the economic feasibility of the concrete. In this paper, the particle size of ceramsite is controlled at 5~20 mm [14,15].

(3) Fine aggregate

Natural river sand is used as the fine aggregate in the test, which is medium sand with good gradation. The performance test method of sand refers to “construction sand” [16]. The bulk density and dry apparent density are 1503 kg/m^3^ and 2627 kg/m^3^, respectively. The fineness modulus is 2.51, and the mud content is 0.7%.

(4) Mineral admixtures

II grade fly ash is used in the test. The ignition loss is 1.62%, the activity index is 75%, and the density is 2200 kg/m^3^. The S95 grade slag is selected, the loss on ignition is 3%, the activity index is 95%, and the density is 2800 kg/m^3^ [17,18].

(5) Fiber

In this paper, PPF is used to prepare fiber ceramsite concrete. The fiber length is 19 mm, the diameter is 31 μm, the tensile strength of the monofilament is 500 MPa, the elastic modulus is 3.5 GPa, and the fracture elongation is 30%.

(6) Admixtures

Sodium gluconate is used as a retarder; the solid content is 99.2% and diluted to 10%. Polycarboxylic acid concentration is used as a water-reducing agent, with a solid content of 40% and a water reduction rate of 30%. Non-ionic polyacrylamide is used as a tackifier [19].

**Figure 1:**
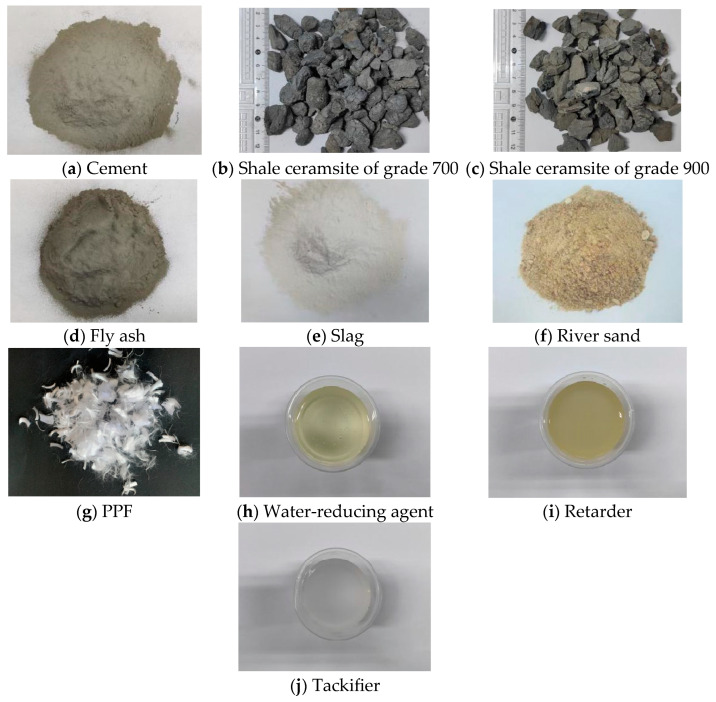
Main raw materials for testing.

Photos of all raw materials are shown in Figure 1.

### 2.2. Preparation Process

**Figure 2:**
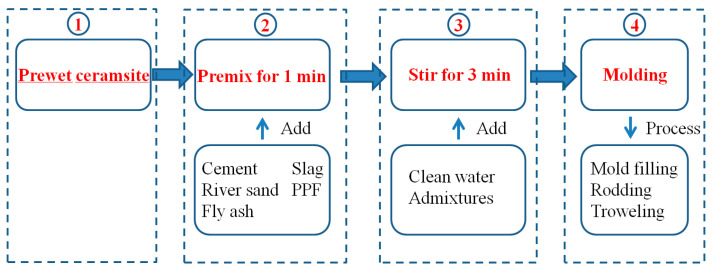
The preparation process of ceramsite concrete.

The density of ceramsite is lower than that of the slurry, which often causes the floating of coarse aggregates and the delamination of the mixture, reducing the uniformity of the concrete. To alleviate this phenomenon, through experimental exploration, this paper optimizes the following four aspects of the preparation process of fiber-reinforced ceramsite concrete. The optimized overall preparation process is shown in Figure 2.

(1) Aggregate prewetting. The ceramsite was soaked 24 h in advance, and the surface moisture was drained 30 min before the preparation of concrete. The test showed that the 24 h soaking can make the ceramsite saturated by water absorption. As a result, the weight of saturated ceramsite increases, reducing the density disparity between the ceramsite and the slurry. At the same time, saturated ceramsite can form a “reservoir” inside the concrete, effectively improving the microstructure of the interface area and reducing the early shrinkage of the concrete [20].
(2) Adjust the sand ratio. An increase in the sand ratio augments the specific surface area of the slurry, resulting in increased slurry viscosity to a certain extent, which hinders the floating of ceramsite. However, an excessive sand ratio will elevate the bulk density of the mortar, increasing the density difference between the aggregate and the mortar, and leading to the segregation of the mixture. Based on the comprehensive dry density, compressive strength, slump, and other indicators of the test, the preferred sand rates for LC20, LC30, and LC40 were 38%, 36%, and 36%, respectively.
(3) Raw material mixing. Firstly, the aggregate, cementitious material, and polypropylene fiber are pre-mixed. The aggregate includes sand and pre-wetted ceramsite, while the cementitious material includes cement, fly ash, and slag. The fiber is dispersed within the aggregate and pre-mixed for 1 min to create a dry mixture. The air-entraining agent is mixed with water and then added to the mixer. The mixture is subjected to 3 min of mixing to produce the concrete mixture. The uniformly small bubbles produced by the air-entraining agent during mixing can reduce the density of the slurry, decrease the floating rate of the aggregate, and also bring the microsphere effect and improve the workability of the mixture [20]. Additionally, the fiber, supported by three-dimensional chaos within the slurry, can increase the resistance of ceramsite to float in the slurry.
(4) Strictly control the length of insertion and vibration. After the ceramsite concrete is poured into the mold, the ceramsite concrete is fully inserted along the four inner walls of the mold, and each inner wall is inserted 7–8 times; after the insertion is completed, the mold is placed on the vibration table for vibration twice, with each vibration lasting for 5 s, and the surface of the specimen is leveled after two vibrations.

**Figure 3:**
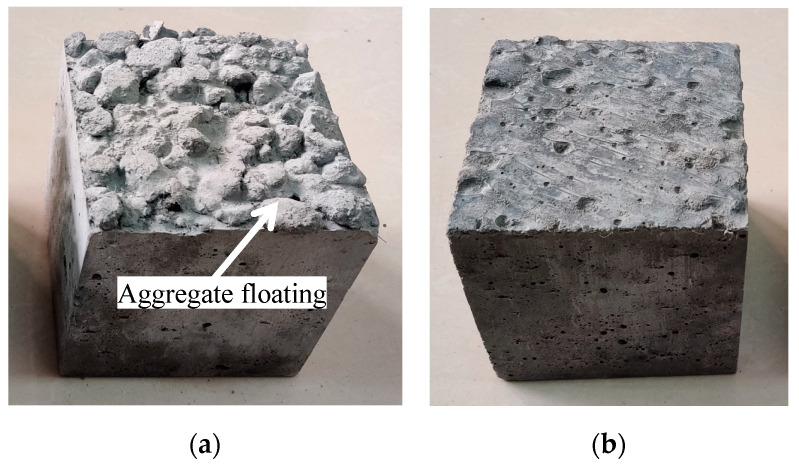
Comparison of specimens before and after the optimization of the preparation process. (**a**) Specimen prepared before the process optimization; (**b**) specimen prepared after the process optimization.

Figure 3 shows the comparison of specimens before and after the optimization of the preparation process. The mineral admixtures, sand ratios, water–binder ratios, and total amounts of cementitious materials of the two test blocks are the same. Among them, Figure 3a shows the specimen prepared before the optimization of the process. It can be seen that the ceramsite floats obviously, and the homogeneity of the specimen is poor. The specimen in Figure 3b is prepared by adding a thickening agent according to the process shown in Figure 1. This effectively improves the “floating” phenomenon of the ceramsite and enhances the uniformity of the fiber-reinforced ceramsite concrete.

## 3. Experimental Study

### 3.1. Specimen Design

The low compressive strength of coarse aggregate results in the difference between the failure mechanism of ceramsite concrete after compression and that of ordinary concrete [21,22]. In order to explore the influence of strength grade and PPF fiber content on the mechanical properties and failure modes of uniaxial compression, six groups of 54 fiber-reinforced ceramsite concrete with three strength grades were prepared. The test design includes varying PPF volume content levels of 0%, 0.05%, 0.075%, and 0.1% for each of the three strength grades, LC20, LC30, and LC40. There are six groups of mix ratios, with each group matched with 9100 mm × 100 mm × 300 mm prism specimens. The parameters of the mix ratio are shown in Table 1. For example, LC20-0.05P indicates LC20 ceramsite concrete with a 0.05% fiber volume content, where the volume content refers to the proportion of the fiber’s volume to the overall mixture component volume [23,24].

### 3.2. Test Method

**Figure 4:**
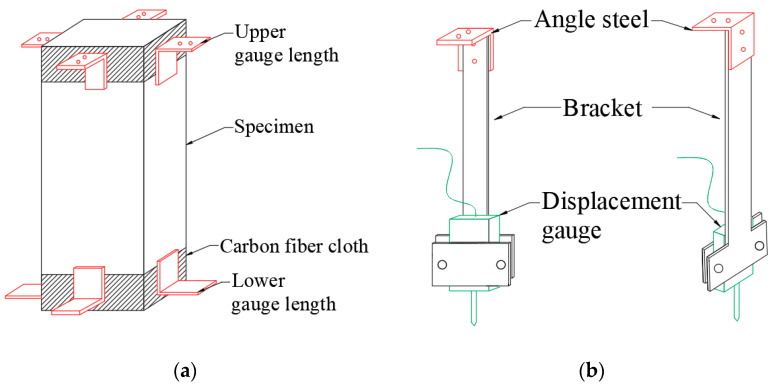

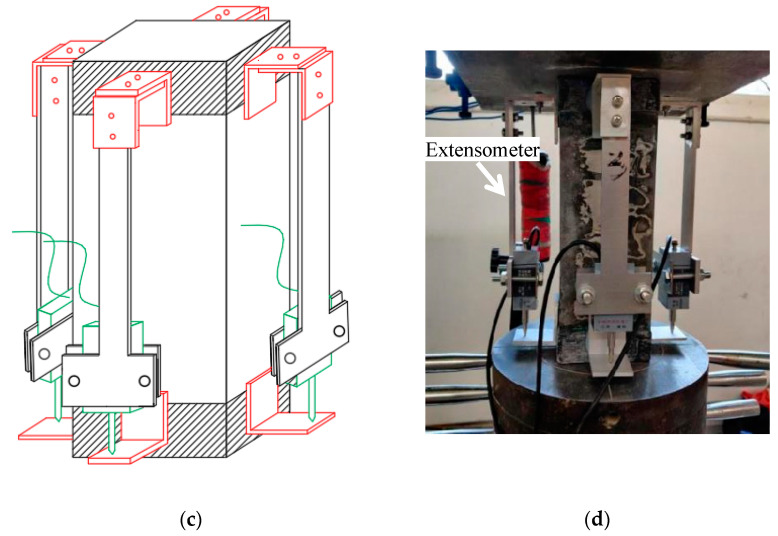
The uniaxial compression stress–strain device diagram. (**a**) Processed diagram of specimen diagram; (**b**) displacement gauge and its bracket; (**c**) overall diagram; (**d**) physical picture.

To prevent the premature failure of the specimens at the areas near the compressed surfaces at the upper and lower ends due to excessive lateral deformation, the sides within a range of 40 mm at the upper and lower ends of the specimens are pre-polished, and three circles of 30-mm-wide carbon fiber cloth are wound and pasted. Aluminum alloy angle steels with a thickness of 4 mm are pasted at the upper and lower ends of the specimens as footmarks, with a spacing of 280 mm between the footmarks, as shown in Figure 4a. The displacement gauge bracket is composed of an angle steel, a T-shaped steel plate, and a rectangular steel plate. The displacement gauge is fixed between the rectangular steel plate and the T-shaped steel plate by two bolts. The upper end of the T-shaped steel plate is fixed to the angle steel by bolts (Figure 4b). The angle steel is fixed to the footmark at the top of the specimen, and the movable rod of the displacement gauge is against the upper surface of the footmark at the bottom of the specimen (Figure 4c). To eliminate the influence of loading eccentricity, displacement gauges are arranged on all four sides of the specimen. This device can measure the relative displacement between two gauge lengths during the loading process. An extensometer is installed between the upper and lower platens of the press to control the displacement loading of the press. The test site is shown in Figure 4d.

The YAW-G3000 high stiffness rock concrete testing machine was used (manufactured by Hangzhou Bangwei Electromechanical Control Engineering Co., Ltd., Hangzhou, China); before the test, the test force control was used for preloading, with a loading rate of 0.3 MPa/s. It was loaded to 20% of the ultimate load, with sustained loading for 60 s, and then it was unloaded to the initial load value of 0.5 MPa, and so repeated three times. Displacement control loading was used for formal loading of the whole process, with the initial loading rate of 0.15 mm/min, after reaching 70% of the peak load to 0.1 mm/min, until the descending section was completed, and press and displacement meter data were recorded.

## 4. Test Results and Analysis

### 4.1. Failure Pattern

**Figure 5:**
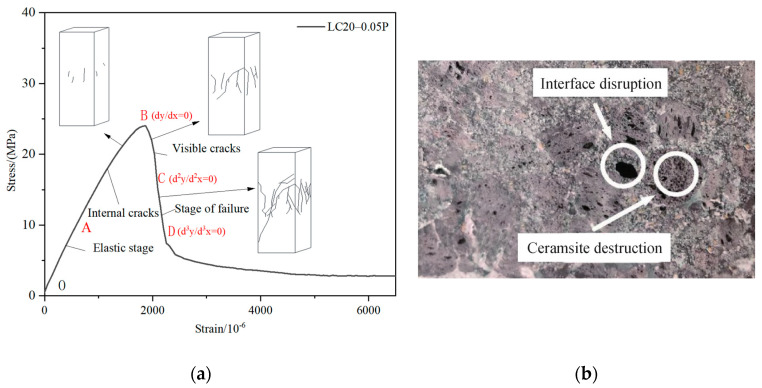
Force characteristics of specimens. (**a**) Complete stress–strain curves of typical specimens; (**b**) typical failure section diagrams of specimens.

Similar to ordinary concrete, the uniaxial compression failure process of fiber ceramsite concrete specimens undergoes four stages (Figure 5), but the failure mechanism and failure phenomena of each stage are different from those of ordinary concrete. The stress and deformation characteristics of each stage are as follows: ① OA elastic stage: the average stress of the section is small (*σ* = (0.35~0.55) *f*_c_), the stress and strain are linear, and no significant macroscopic deformation is observed; ② AB internal crack development stage: with the increase in load, the specimen emits gap, a subtle “crackle” sound, and there is no crack on the surface of the specimen, while the slope of the curve is smaller than that of the OA section, which may be caused by the development of microcracks in the specimen; ③ BC visible crack development stage: the curve reaches the peak stress at point B. Beyond point B, the first visible crack emerges to the specimen surfaces, and then several cracks develop rapidly, accompanied by the spalling of surface concrete debris. As the strain continues to increase, the stress decreases sharply; ④ CD failure stage: with the continuous increase in strain, the rate of stress decline slows down, and the curve approaches a horizontal trend. At this time, the bearing capacity is provided by the residual bonding force and friction resistance between cracks, and the cracks penetrate the specimen.

As can be seen from the failure cross-section shown in Figure 5b, the cracking failure of the ceramsite concrete specimens is mainly caused by the failure of ceramsite and interfacial failure, while the failure of ordinary concrete usually only shows the failure of the interface between aggregates and cement paste. The strength of ceramsite is lower than that of natural stones. During the compression process, a large number of cracks develop through the ceramsite, resulting in the fracture of ceramsite and the failure of the specimens. At the same time, some small holes can also be observed in the failure cross-section, which is a phenomenon of interfacial failure. Some initial cracks develop around the ceramsite and pass through the weak connection interface between the ceramsite and the cement matrix, leading to the failure of the specimens.

The comparative test results show that there are some differences in the uniaxial compression failure stages of ceramsite concrete with different strength grades and fiber contents (Figure 5).

**Figure 6:**
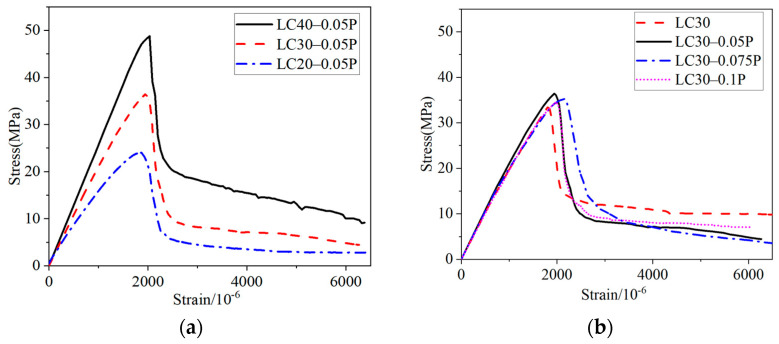
Comparison of stress–strain curves of fiber-reinforced ceramsite concrete under uniaxial compression. (**a**) The effect of strength grade; (**b**) the influence of fiber content.

**Figure 7:**
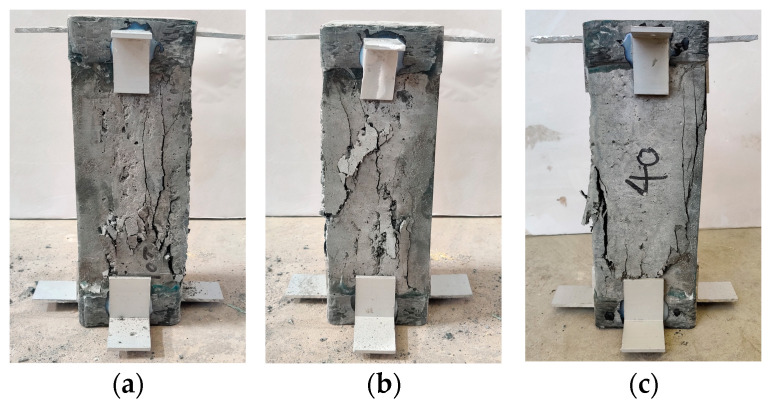
Failure modes of fiber-reinforced ceramsite concrete with different strengths under uniaxial compression. (**a**) LC20; (**b**) LC30; (**c**) LC40.

Figure 6a shows that in the initial stage of loading, high-strength concrete exhibits a longer linear elastic stage compared with low-strength concrete, the elastic limit of LC40 can reach about 48.8 MPa, and the slope of the rising section of the curve is the steepest, while the elastic limit of LC20 is about 24.0 MPa. After the curve enters the descending section, the surface cracks of low-strength specimens appear earlier, and the cracks are finer and more, while the cracks of high-strength specimens are less. With the increase in strength, the cracks develop more rapidly, and the descending section of the curve is steeper. When the high-strength concrete is destroyed, it exhibits 1~2 penetrating cracks, while the low-strength concrete, in addition to the penetrating cracks, features multiple discontinuous fine cracks, indicating that the higher the strength, the greater the brittleness. Figure 7 depicts the uniaxial compression failure patterns of fiber-reinforced ceramsite concrete with different strengths.

**Figure 8:**
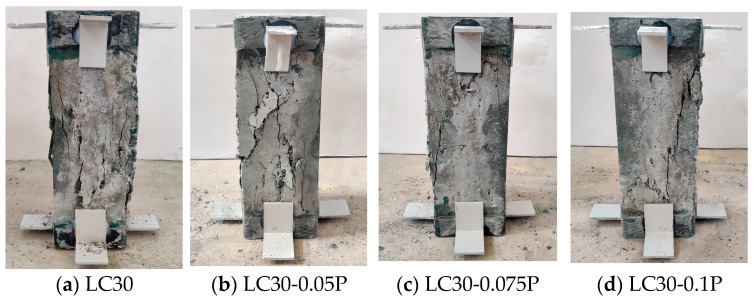
Failure modes of fiber-reinforced ceramsite concrete with different fiber content under uniaxial compression. (**a**) LC30; (**b**) LC30-0.05P; (**c**) LC30-0.075P; (**d**) LC30-0.1P.

Figure 6b shows that the compressive failure of ceramsite concrete with different fiber content also comprises four stages, but there are slight differences. The addition of fiber has little effect on the initial elastic modulus and elastic limit of concrete, while the peak stress is improved. After entering the stage of internal crack development, the internal crushing sound of the specimen with PPF is slightly reduced. After the curve enters the descending section, with the increase in PPF content, the time of cracks on the surface of the specimen is slightly delayed, and the number of cracks and the falling concrete fragments are also relatively reduced. Figure 8 illustrates the uniaxial compression failure pattern of fiber-reinforced ceramsite concrete with different fiber content based on LC30. The above phenomenon is mainly because the fiber is supported by three-dimensional chaos in the hardened paste, which improves the strength of the hardened paste. When the microcracks in the hardened paste extend to the fiber, the fiber is gradually in a debonding state after being pulled, leading to the formation of gaps at the interface between the hardened paste and the fiber. This gap delays the development of internal microcracks. After the curve passes the peak point, the concrete crack gradually develops into a visible crack, and a bond slip occurs between the fiber and the hardened slurry. The tensile stress of the crack section is mostly provided by the fiber, which delays the development of the crack and improves the ductility of the concrete [25].

### 4.2. Analysis of the Influencing Factors of the Characteristic Points of the Stress–Strain Curve

The shape of the stress–strain curve reflects the uniaxial compression failure process of lightweight aggregate concrete, and the characteristic points on the curve offer insights into the stress state and deformation characteristics of concrete. Table 2 is a summary of the characteristic points of the stress–strain full curve of fiber-reinforced ceramsite concrete, and the values in the table are the average values of the test values of the six groups of specimens. Among them, the peak strain (*ε*_c_) corresponds to the strain at which the peak stress (*σ*_c_) is achieved, the ultimate strain (*ε*_0.5_) corresponds to the strain at which the stress in the descending section of the curve drops to 50% of the peak stress (*σ*_c_), the initial elastic modulus *E*_0_ is the corresponding secant slope when the stress of the ascending section of the curve is 0.4*σ*_c_, and the secant modulus *E*_c_ is the corresponding secant slope of the curve at the peak stress *σ*_c_.

#### 4.2.1. Peak Strain and Ultimate Strain

**Figure 9:**
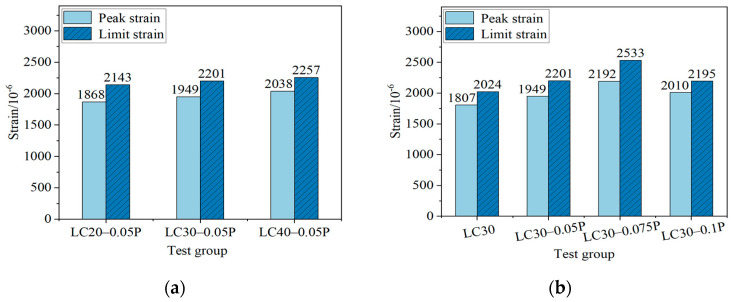
The influence of various factors on *ε*_c_ and *ε*_0.5_. (**a**) The effect of strength grade; (**b**) the influence of fiber content.

Figure 9 is the influence of different strength grades and different fiber contents on the characteristic points *ε*_c_ and *ε*_0.5_ of the uniaxial compressive stress–strain curve of fiber-reinforced ceramsite concrete. Among them, the compressive strength of LC20-0.05P is 24 MPa; in the LC30 group, LC30-0.05P has the highest compressive strength, reaching 36.4 MPa; and the compressive strength of LC40-0.05P is 48.8 MPa. Comparing Figure 9a, it is found that compared with LC20-0.05P, as the strength grade increases, *ε*_c_ is increased by 4.3% and 9.1%, *ε*_0.5_ is increased by 2.7% and 5.3%, while the difference between *ε*_c_ and *ε*_0.5_ decreases by 8.4% and 20.4%. Comparing Table 2 and Figure 9b, it is found that for LC30 ceramsite concrete with PPF contents of 0.05%, 0.075%, and 0.1%, compared with the benchmark group LC30, σc increased by 9.0%, 6.0%, and 4.2%; *ε*_c_ increased by 7.9%, 21.3%, and 11.2%; and *ε*_0.5_ increased by 8.7%, 25.1%, and 8.4%, while the difference between *ε*_c_ and *ε*_0.5_ increased initially and then decreased, with an increase of 16.1%, 57.1%, and 14.7%, respectively.

Similar to ordinary concrete, the *ε*_c_ and *ε*_0.5_ values of fiber ceramsite concrete with higher strength are also higher. Correspondingly, the descending section is steeper, indicating that the brittleness of fiber ceramsite concrete increases with the increase in strength. The contribution of PPF to the increase in *ε*_c_ and *ε*_0.5_ indicates that the crack resistance of PPF is effective, with the best effect at a fiber content of 0.075%. The improvement of the ductility of ceramsite concrete by fiber is mainly achieved by the absorption of tensile stress by fiber. At the initial stage of loading, the fiber exhibits a good bonding effect with the cement matrix and bears the stress together with the matrix and aggregate. With the increase in load, microcracks begin to appear in the matrix, and the fiber is gradually in a state of debonding after being pulled, blocking the extension of cracks. The fine cracks inside the concrete gradually develop, and the bond slip occurs between the fiber and the matrix to absorb the internal tensile stress. At this point, the cracks continue to develop across the fiber, and most of the tensile stress carried by the matrix is provided by the fiber, which enhances the deformation performance of the ceramsite concrete after reaching the peak stress. However, when the fiber is pulled off or pulled out, the improvement effect of fiber on the brittleness of concrete gradually fails [26].

#### 4.2.2. Initial Elastic Modulus and Secant Modulus

**Figure 10:**
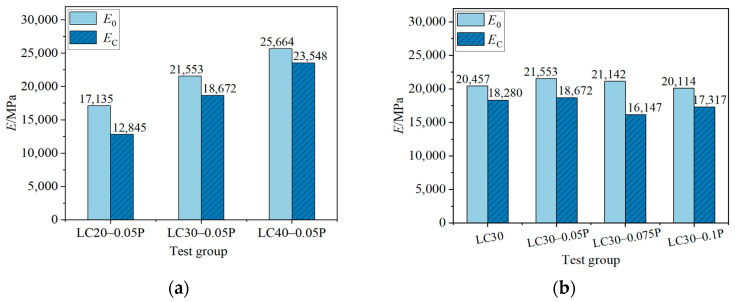
The influence of various factors on *E*_0_ and *E*_c_. (**a**) The effect of strength grade; (**b**) the influence of fiber content.

Figure 10 is the influence of different strength grades and different fiber contents on the initial elastic modulus *E*_0_ and the secant elastic modulus *E*_c_ of the characteristic points of the uniaxial compressive stress–strain curve of fiber-reinforced ceramsite concrete. Comparing Figure 10a, it is found that the *E*_0_ and *E*_c_ of fiber-reinforced concrete are smaller than those of ordinary concrete of the same grade; compared with LC20-0.05P, as strength grade increases, *E*_0_ increases by 25.8% and 49.8%, and *E*_c_ increases by 45.3% and 83.3%, while the difference between *E*_0_ and *E*_c_ showed a decreasing trend. Comparing Figure 10b, it is found that *E*_0_ and *E*_c_ are not sensitive to fiber content, but the difference between the two is significantly improved. For LC30 ceramsite concrete with PPF contents of 0.05%, 0.075%, and 0.1%, compared with the benchmark group LC30, the difference between *E*_0_ and *E*_c_ is increased by 32.3%, 129.4%, and 28.5%, respectively, indicating that the addition of PPF contributes to improving the plastic performance of the rising stage of the curve.

The elastic modulus of fiber ceramsite concrete is lower than that of ordinary concrete, which is mainly attributed to the difference of the failure mode between them. The strength of ceramsite is much smaller than that of ordinary gravel. The failure of ordinary concrete mainly comes from the failure of a weak interface between cement stone and aggregate, while the failure of ceramsite concrete mainly results from the failure of aggregate and a weak interface. At the same time, both *E*_0_ and *E*_c_ increase with the increase in strength grade, which is mainly due to the increase in strength and density, but the difference between them decreases with the increase in strength grade, indicating that the higher the strength grade, the more significant the linear characteristics of the rising section of the curve. The elastic modulus of PPF is much smaller than that of concrete. According to the theory of composite materials, the elastic modulus of composite concrete will decrease. However, due to the small amount of fiber, the fiber content has little effect on the elastic modulus of concrete. With the increase in fiber content, *E*_c_ fluctuates significantly, and the overall trend decreases initially and then increases. The main reason is that the increase in fiber improves the deformation performance of ceramsite concrete before failure, subsequently resulting in changes in *ε*_c_ and consequently bringing about the change of *E*_c_.

## 5. Uniaxial Compression Stress–Strain Relationship Model

### 5.1. Comparative Analysis of Models

In recent years, scholars at home and abroad have conducted extensive and in-depth research on the mechanical properties of lightweight aggregate concrete. However, there is still no consensus on the expression of its uniaxial compression constitutive model. This is because the coarse aggregates of lightweight aggregate concrete are significantly different from those of ordinary concrete, and most of the existing models are based on the regression analysis of test data of ordinary concrete. Therefore, it is difficult to accurately describe the failure characteristics of lightweight aggregate concrete. Lin H J et al. [10] fitted and modified the test curves through five existing constitutive models and proposed a mathematical model for the uniaxial compression stress–strain curve of high-toughness polypropylene (HTPP) fiber-reinforced ceramsite concrete, which was in good agreement with the test results. Given that the research object of this paper is similar to that of Lin et al., and the method they adopted has been verified, this paper, based on referring to the source of their model, selects six typical constitutive models for comparative analysis, as shown in Table 3. Among them, Carreira et al. [27] adopted a full-segment model, while Guo et al. [28], Wang Z Y et al. [29], Yang et al. [30], Wang H L et al. [31], and Zhao S B et al. [32] adopted segmented models. Guo’s research object was ordinary concrete, while the research objects of the other five people covered lightweight aggregate concrete.

The Wang Z Y model takes into account the large brittleness of LAC and the steep descending section of the stress–strain curve. It refines the descending-section model into two sub-segments, which is applicable to LAC with a strength of LC30-LC55, using volcanic rock ceramsite, calcined clay ceramsite, and crushed shale ceramsite as lightweight aggregates. The Carreira model is applicable to ordinary concrete with a strength of 8 MPa–140 MPa and lightweight aggregate concrete with a strength of 23 MPa–80 MPa. The Wang H L model is applicable to all-lightweight and semi-lightweight aggregate concrete of LC50, where the coarse aggregates are fly-ash ceramsite and shale ceramsite, the fine aggregates are shale lightweight sand and natural river sand, and the fibers are steel fibers. The Zhao model is applicable to steel-fiber-reinforced all-lightweight concrete of LC40, where the coarse aggregates are shale ceramsite with a size of 2 mm–20 mm, the fine aggregates are shale lightweight sand with a size of 1.6 mm–5 mm, and the fibers are thin-plate-sheared and indented steel fibers with a length of 36.68 mm and a diameter of 1.35 mm. The Yang model, based on the Carreira model, assigns different meanings and values to the parameter β in the ascending and descending sections, and is applicable to concrete with a strength of 10–170 MPa and a density of 1200–4500 kg/m^3^. The research object of this paper is fiber-reinforced ceramsite concrete with strengths of LC20, LC30, and LC40. The concrete strength and type are close to the applicable ranges of the above-mentioned research results. Therefore, the above-mentioned models have a certain reference significance.

**Figure 11:**
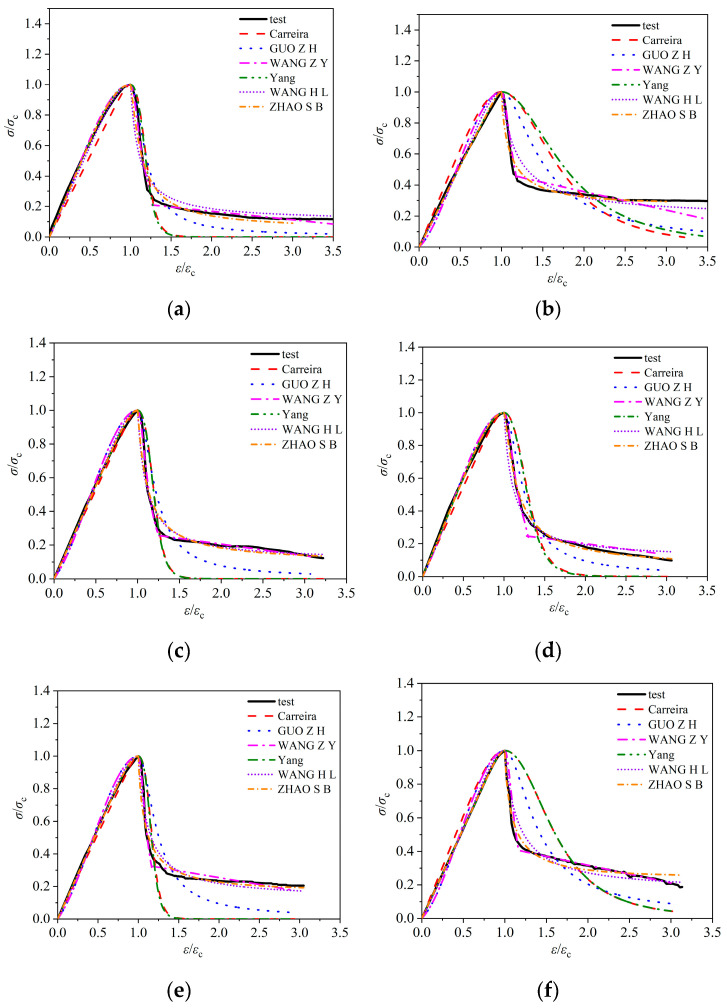
The comparison between fitting model curve and test curve. (Remark: y = *σ*/*σ*_c_, *σ* is stress, *σ*_c_ is peak stress, x = *ε*/*ε*_c_, *ε* is strain, *ε*_c_ is peak strain.) (**a**) LC20-0.05P; (**b**) LC30; (**c**) LC30-0.05P; (**d**) LC30-0.075P; (**e**) LC30-0.1P; (**f**) LC40-0.05P.

**Figure 12:**
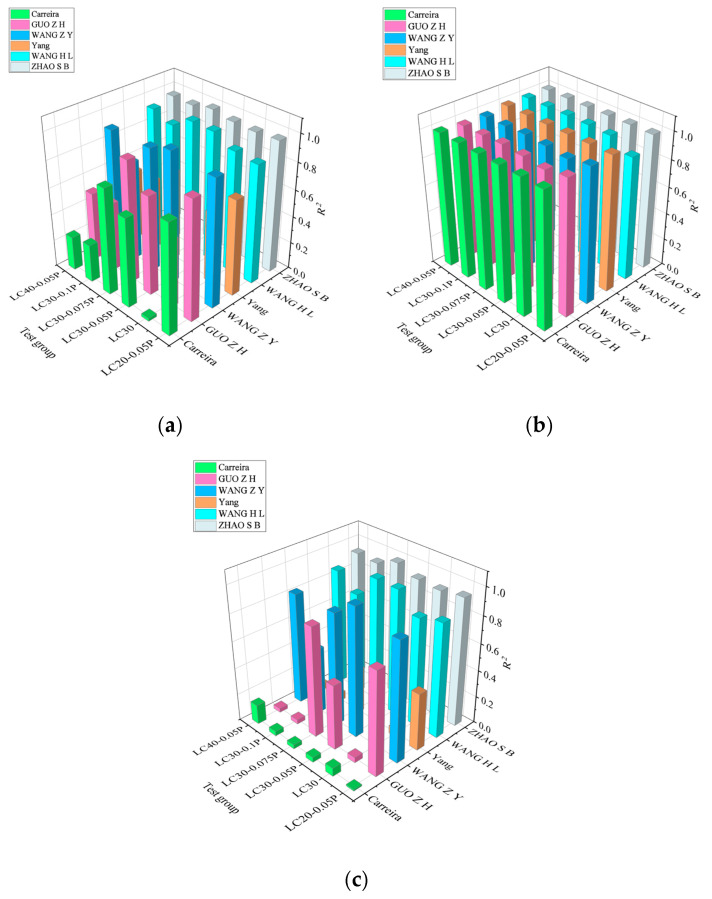
The summary of fitting degree R^2^ between model formula and test curve. (**a**) Full curve fitting R^2^; (**b**) ascending fitting degree R^2^; (**c**) falling segment fitting degree R^2^.

The characteristic points of the measured curves of the six groups of working conditions in this paper are substituted into the above six models, respectively, and the corresponding model curves are obtained and then compared with the test curves. The results are shown in Figure 11, and the fitting degree of each model curve and the test curve is shown in Figure 12.

The comparative analysis of the above fitting results shows that the six models have good fitting results for the rising section of the curve, with R^2^ values above 0.98. This is mainly due to the fact that the ceramsite concrete is in the elastic stage and the internal crack development stage during the rising section of the curve. At this point, the macroscopic cracks of the specimen have not yet appeared, and the concrete category has little effect on the shape of the dimensionless curve. Therefore, the six models yield good fitting results for the rising section of the curve. Conversely, the fitting results of the six models for the descending section of the curve are quite different, mainly due to the different brittleness of different types of concrete in the visible crack development stage, leading to differences in the shape of the dimensionless curve. The addition of fiber improves the brittleness of ceramsite concrete, resulting in a gentle shape for the descending section of the curve. Therefore, there are also differences in the fitting results of the stress–strain full curve of the fiber-reinforced ceramsite concrete by the six models, mainly stemming from the large difference in the aggregate of the concrete corresponding to each model. Based on the comprehensive comparison results, Zhao’s model provides the best fit for the ascending section, the descending section, and the entire curve of the test results, mainly because the model and this paper use ceramsite as coarse aggregate, and the concrete strength grade is similar.

### 5.2. Model Updating

**Figure 13:**
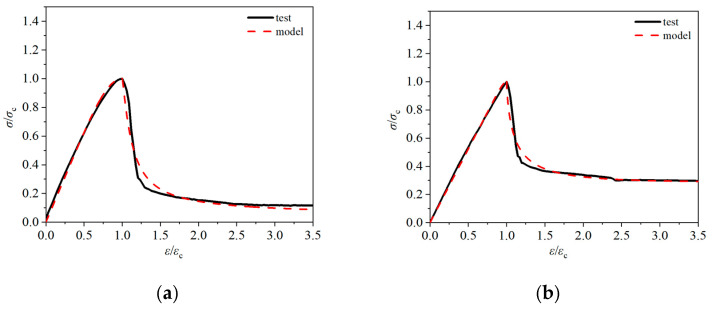

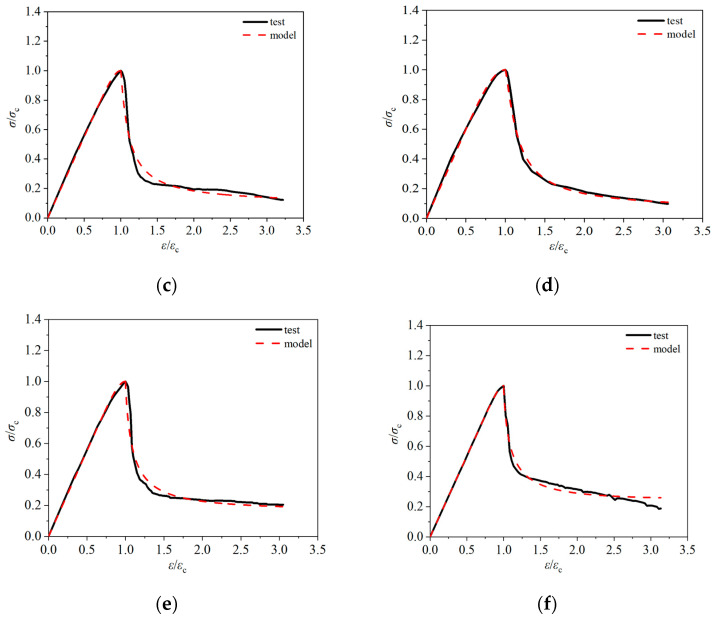
The comparison between modified model curve and test curve. (**a**) LC20-0.05P; (**b**) LC30; (**c**) LC30-0.05P; (**d**) LC30-0.075P; (**e**) LC30-0.1P; (**f**) LC40-0.05P.

Comparing the measured curves of six groups of working conditions with the fitting results of the above six models, considering the mechanical characteristics of fiber-reinforced ceramsite concrete, the stress–strain curve expression of fiber-reinforced ceramsite concrete in this paper adopts the segmented formula, and the rising and falling segments of the stress–strain curve adopt Zhao’s model; see Formula (1). However, because Zhao’s model is aimed at steel fiber ceramsite concrete, the deformation performance of the curve descending section is different from that of the concrete in this paper. Therefore, based on the measured values, the shape correlation coefficients αc and b in the curve descending section of the model are modified, leading to the proposal of the stress–strain correction model based on fiber-reinforced ceramsite concrete. The shape correlation coefficients αc and b in the descending section of the fitted curve and the fitting degree of each section of the curve are shown in Table 4, and the modified model curve is shown in Figure 13.

(1) $$y=\left\{\begin{array}{l} nx/(n-1+x^{n}),x\leq 1\\ x/\left[\alpha_{c}{\left(x-1\right)}^{b}+x\right],x>1 \end{array}\right.$$

In the formula: y = *σ*/*σ*_c_, *σ* is stress, *σ*_c_ is peak stress, x = *ε*/*ε*_c_, *ε* is strain, *ε*_c_ is peak strain, *n* = *E*_c_*ε*_c_/(*E*_c_*ε*_c_ − *σ*_c_), and *E*_c_ is elastic modulus.

It can be seen from Table 4 and Figure 13 that the modified model fits well with the experimental values. The average R^2^ values of ascending, descending, and full curve fitting are 0.9979, 0.9425, and 0.9702, respectively. The modified model has the best fitting result for LC30-0.075P, with an R^2^ value of 0.9927, while the fitting result of LC30 without fiber is poor. This suggests that the modified model proposed in this paper has certain applicability to the prediction of the stress–strain full curve relationship of fiber-reinforced ceramsite concrete under uniaxial compression.

## 6. Conclusions

In this paper, by adding polypropylene fibers and optimizing the preparation process, the compressive performance of ceramsite concrete has been improved, which can provide a reference for promoting the application of ceramsite concrete in engineering. Through uniaxial compressive tests, this paper explores the mechanical properties and failure modes of fiber-reinforced ceramsite concrete, with a focus on the influence of concrete strength and fiber content on its stress–strain relationship, and proposes a model expression for the stress–strain curve of fiber-reinforced ceramsite concrete under uniaxial compression. The specific conclusions are as follows:

(1) The preparation process of fiber-reinforced ceramsite concrete has been optimized. Based on the requirement of the uniformity of ceramsite concrete mixtures, by controlling the particle size and shape of ceramsite; pre-wetting the ceramsite; adding mineral admixtures, water-reducing agents, thickening agents, and polypropylene fibers; and adjusting the vibration-forming process, the problem of “aggregate floating” in ceramsite concrete has been effectively improved, and the material uniformity has been enhanced.
(2) As the polypropylene fiber content increases, the peak stress *σ*_c_ in the compressive stress–strain curve of ceramsite concrete is increased to a certain extent. It reaches its peak when the fiber volume content is 0.05%, with an increase of 8.98%. The difference between the initial elastic modulus *E*_0_ and the secant modulus *E*_c_ increases, and the slope of the descending section of the curve becomes significantly gentler. When the fiber volume content is 0.075%, the peak strain *ε*_c_ and the ultimate strain *ε*_0.5_ increase by 21.3% and 25.2%, respectively. This indicates that an appropriate PPF content can better improve the deformation performance of ceramsite concrete before failure, enabling it to have better toughness during the loading process and effectively reducing the risk of brittle failure.
(3) With the increase in the strength grade of PPF-ceramsite concrete, the linear characteristic of the ascending section of the stress–strain curve becomes more significant, the linear-elastic stage is extended, and *ε*_c_ increases. However, at the same time, the slope of the descending section also increases. *E*_0_ and *E*_c_ increase slightly, and the difference between them gradually decreases. The difference between *ε*_0.5_ and *ε*_c_ decreases. This shows that as the strength grade increases, the brittleness of the specimens becomes more obvious.
(4) Based on the measured values, after fitting and comparing with the stress–strain model formulas proposed by six scholars at home and abroad, the model curve of Zhao was selected, and the shape coefficient of its descending section was modified. Subsequently, a segmented correction model expression for the uniaxial compressive stress–strain curve of PPF-ceramsite concrete studied in this paper was proposed. The modified model has a good fit with the full experimental curve of this paper, which can provide an accurate theoretical reference for the deformation research of fiber-reinforced ceramsite concrete components and structures under external loads, and promote the application and development of this material in engineering practice.

In order to enhance the understanding of PPF-ceramsite concrete and promote its wide application in practical engineering, the project team will conduct further research in the following aspects. On the one hand, the actual engineering environment is complex and changeable. In the future, the influence of different environmental factors on the durability of PPF-ceramsite concrete can be studied in depth. Secondly, through methods such as electron microscope scanning, the occurrence and development of internal cracks in PPF-ceramsite concrete at the micro-level, as well as the synergistic strengthening mechanism of ceramsite and PPF, can be studied to further optimize the mechanical properties of ceramsite concrete.

## Figures and Tables

**Table 1:** Design table of mix ratio of cube compression specimen.

Specimen	Cement/kg	Fly Ash/kg	Slag/kg	River Sand/kg	Ceramsite/kg	Water /kg	Water Reducer /%	PPF /%	Retarder /%	Viscosifier/kg
LC20-0.05P	315	67.5	67.5	709	536	126	0.93	0.05	0.1	0.21
LC30	425	37.5	37.5	661	545	125	0.60	0	0.1	0.21
LC30-0.05P	425	37.5	37.5	661	545	125	0.96	0.05	0.1	0.21
LC30-0.075P	425	37.5	37.5	661	545	125	1.10	0.075	0.1	0.21
LC30-0.1P	425	37.5	37.5	661	545	125	1.35	0.1	0.1	0.21
LC40-0.05P	425	37.5	37.5	671	788	115	1.25	0.05	0.1	0

**Table 2:** The summary of feature points of the stress–strain curve.

Specimen	Peak Stress *σ*_c_ (MPa)	Peak Strain *ε*_c_ (×10^−6^)	Limit Strain *ε*_0.5_ (×10^−6^)	Initial Elastic Modulus *E*_0_ (MPa)	Secant Modulus *E*_c_ (MPa)
LC20-0.05P	24.0	1868	2143	17135	12845
LC30	33.4	1807	2024	20457	18280
LC30-0.05P	36.4	1949	2201	21553	18672
LC30-0.075P	35.4	2192	2533	21142	16147
LC30-0.1P	34.8	2010	2195	20114	17317
LC40-0.05P	48.8	2038	2257	25664	23548

**Table 3:** The comparison of stress–strain relationship models of concrete under uniaxial compression.

Number	Investigator	Expression	Parameter Declaration
1	Carreira	$y=\frac{\beta x}{\beta -1+x^{\beta }}$	*β* = 1/[1 − (*f*_c_/*ε*_c_*E*_c_)], *E*_c_ = 10,200 (*f*_c_)^1/3^, *ε*_c_ = (0.71 *f*_c_^’^ + 168) × 10^−5^, *f*_c_ is the axial compressive strength of concrete.
2	GUO Z H	$y=\left\{\begin{array}{l} \alpha_{a}x+(3-2\alpha_{a})x^{2}+(\alpha_{a}-2)x^{3},x\leq 1\\ \frac{x}{\alpha_{d}{\left(x-1\right)}^{2}+x},x\geq 1 \end{array}\right.$	*α*_a_ is the ratio of initial elastic modulus to peak secant modulus; *α*_d_ is related to concrete strength grade and restraint mode, *α*_d_ ≥ 0.
3	WANG Z Y	$y=\left\{\begin{array}{l} \alpha_{a}x+(3-2\alpha_{a})x^{2}+(\alpha_{a}-2)x^{3},x\leq 1\\ \frac{x}{\alpha_{d1}{\left(x-1\right)}^{2}+x},1\leq x\leq 1.25\\ \alpha_{d2}(5-x),x\geq 1.25 \end{array}\right.$	*a*_a_ is the ratio of initial elastic modulus to peak secant modulus; *α*_d1_, *α*_d2_ is related to concrete strength grade and constraint mode.
4	Yang	$y=\frac{(\beta_{1}+1)x}{\beta_{1}+x^{\beta_{1}+1}}$	$\mathrm{Climb}\;\mathrm{element}:\beta_{1}=0.20\exp\left[0.73{\left(10/f_{c}^{’}\right)}^{0.67}{\left(\rho /2300\right)}^{1.17}\right]$ $\mathrm{Sloping}\;\mathrm{portion}:\beta_{1}=0.41\exp\left[0.77{\left(10/f_{c}^{’}\right)}^{0.67}{\left(\rho /2300\right)}^{1.17}\right]$
5	WANG H L	$y=\left\{\begin{array}{l} \alpha x+(5-4\alpha )x^{4}+(3\alpha -4)x^{5},x\leq 1\\ \frac{x}{\beta (x-1)+x},x>1 \end{array}\right.$	*α* is the ratio of initial elastic modulus to secant elastic modulus; *β* reflects the trend of the descending section. The larger the value, the steeper the curve.
6	ZHAO S B	$y=\left\{\begin{array}{l} nx/(n-1+x^{n}),x\leq 1\\ x/\left[\alpha_{c}{\left(x-1\right)}^{b}+x\right],x>1 \end{array}\right.$	*n* = *E*_c_*ε*_c_/(*E*_c_*ε*_c_ − *σ*_c_), *E*_c_ is elastic modulus, *α*_c_ and *b* is the shape coefficient related to the shape of the descending curve.

**Table 4:** The summary of modified model parameters.

Specimen Group	Ascending Section of Curve		Curve Descending Section			Complete Curves
	*n*	R^2^	*α*_c_	*b*	R^2^	R^2^
LC20-0.05P	4.799	0.9956	12.5046	1.2760	0.9459	0.9708
LC30	23.0821	0.9979	4.1828	0.7870	0.9241	0.9610
LC30-0.05P	10.5419	0.9982	8.9121	1.0546	0.9356	0.9669
LC30-0.075P	5.9482	0.9973	9.9690	1.2769	0.9881	0.9927
LC30-0.1P	10.3536	0.9988	6.8221	0.8790	0.9250	0.9619
LC40-0.05P	14.9404	0.9997	4.9135	0.7958	0.9361	0.9679

## Data Availability

All relevant data are within the paper.
